# Test-retest reliability, internal consistency, construct validity and factor structure of a falls risk perception questionnaire in older adults with type 2 diabetes mellitus: a prospective cohort study

**DOI:** 10.1186/s40945-019-0065-4

**Published:** 2019-12-02

**Authors:** Janelle Gravesande, Julie Richardson, Lauren Griffith, Fran Scott

**Affiliations:** 1 School of Rehabilitation Science, McMaster University, Room 403, 1400 Main St., W. Hamilton, ON L8S 1C7 Canada; 2 Department of Health Research Methods, Evidence and Impact, McMaster University, 1280 Main St., W. Hamilton, ON L8S 4K1 Canada

**Keywords:** Balance, Diabetes, Fall risk, Risk perception, Older adults

## Abstract

**Background:**

Older adults with type 2 diabetes (DM2) are at increased risk of falling due to complications including: diabetic peripheral neuropathy, diabetic retinopathy, autonomic neuropathy and diabetic foot ulcers. The purpose of this study was to determine the test-retest reliability, internal consistency, construct validity and to perform factor analysis of a new falls Risk Perception Questionnaire (RPQ) in older community-dwelling adults with DM2.

**Methods:**

A prospective cohort of 30 community-dwelling older adults, ≥ 55 years, with DM2 was assembled. At baseline, perceived risk of falling, fear of falling and physical activity were measured. At time 2 (T2), at least 2 days later, perceived risk of falling was assessed again to determine the test-retest reliability of the RPQ. At time 3 (T3), approximately six weeks later, and time 4 (T4), at least 2 days after T3, perceived risk of falling was assessed by phone to determine the test-retest reliability of the RPQ when administered by phone.

**Results:**

The RPQ demonstrated excellent test-retest reliability when delivered in person (ICC = 0.78, 95% Confidence Interval, CI: 0.59–0.89) and by phone (ICC = 0.82, 95% CI: 0.65–0.91), good internal consistency (α = 0.78) and adequate construct validity (r = 0.52, 95% CI: 0.20–0.74, *p* = 0.003) in older adults with DM2.

**Conclusion:**

Given the good psychometric properties in this sample of persons with Diabetes, the RPQ has the potential to be used in clinical practice as a risk assessment and fall prevention tool. However, further testing needs to be done using a larger sample.

## Background

Older adults with DM2 are at increased risk of falling due to complications including: diabetic peripheral neuropathy, diabetic retinopathy, autonomic neuropathy and diabetic foot ulcers [1]. A study of older adults with diabetic peripheral neuropathy (DPN) (*n* = 30) revealed that individuals with DPN had reduced walking speed, step length and cadence as well as impaired balance, peripheral sensation and reaction time compared to age-matched controls without DM2 [2]. Research also shows that women with diabetes are 1.6 times more likely to have experienced a fall in the past year and are twice as likely to be injured when they fall compared to women without diabetes [3]. Additionally, older adults with DM2 are at increased risk of falling due to cognitive decline [4]. A meta-analysis of 19 studies revealed that individuals with DM2 are at increased risk for vascular dementia (RR = 2.48) [4]. Risk of falling is often measured using balance assessments such as the Berg Balance Test. Another component of risk that is less frequently measured is perception. Risk perception (perceived risk) is a multi-dimensional concept that considers awareness and judgements about the probability of an outcome (e.g. a fall) and its potential consequences as well as judgments about the importance of the risk to the individual [5]. Risk perception research has focused on people’s perceptions of environmental, technological and nuclear risks [6–11]. However, it has played only a small role in health care research [12–14]. Risk perception is important because it helps to predict health behavior [12–14]. A review of older adults’ perceptions, beliefs and behaviors regarding fall prevention revealed that some older adults do not believe they are at risk of falling because they feel healthy and confident [15]. Older adults also believe that external factors such as environmental hazards cause more falls than internal factors such as dizziness or muscle weakness [15]. There is evidence that suggests older adults’ perceptions about falling can be altered which in turn changes their attitudes toward fall prevention. Hughes et al. (2008) assessed fear of falling in older adults (*n* = 3202); individuals from the fall prevention program were less likely than individuals from the comparison group to agree with the statement “older people fall, and there is nothing that can be done about it,” χ^2^ = 17.1, *p* < 0.001 [16]. Additionally, individuals from the fall prevention program were more likely to rate fall prevention as a high or very high priority compared to individuals from the comparison group (χ^2^ = 11.4, *p* < 0.01) [16]. These findings suggest that increasing individuals’ awareness about their risk of falling may result in greater engagement in fall prevention behaviors.

### Development of a risk perception questionnaire for falling

**Fig 1:**
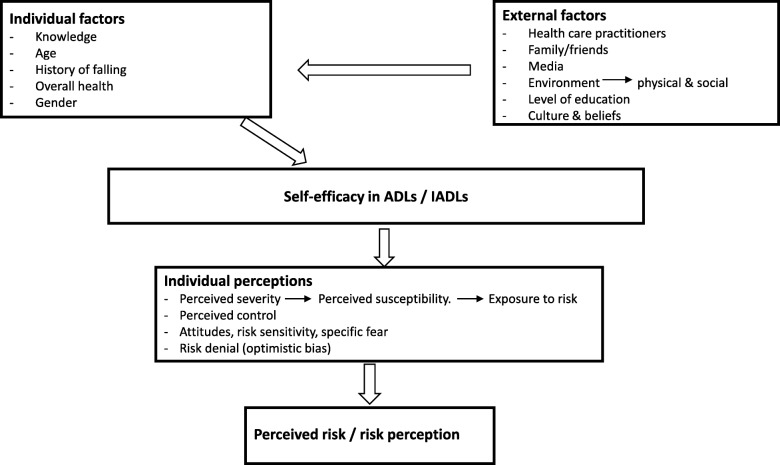
Risk perception model based on the Health Belief Model

A falls Risk Perception Questionnaire (RPQ) was developed using a conceptual model based on the Health Belief Model and the risk perception literature. According to this conceptual model, risk perception/ perceived risk consists of several interacting factors including: external factors, individual factors, self- efficacy in activities of daily living (ADLs) and instrumental activities of daily living (IADLs) as well as individual perceptions (Fig. 1). External factors include: health care practitioners [17], family/friends, media, social environment [18], physical environment [19], level of education [20], culture [21] and beliefs [11]. Individual factors include: knowledge [10], age, gender [20], history of falling and self-rated health [16]. Self-efficacy refers to one’s beliefs about their ability to determine their own behavior and the events that shape their lives [22]. Activities of daily living are common everyday activities including: bathing, dressing and eating [23]. Instrumental activities of daily living are more challenging activities including: managing personal finances and preparing meals [23]. A prospective cohort study of older adults (*n* = 528) found that individuals with poorer fall-related self-efficacy (Tinetti’s Falls Efficacy Scale score ≤ 75) were more likely to experience declines in their ability to perform ADLs (change in ADL score = − 0.829, p< 0.001); these individuals were also at increased risk for subsequent falls (HR = 2.09 (1.31–3.33)) [24]. Individual perceptions include: *perceived severity* (feelings about the seriousness of a disease) [25] and *perceived control* (beliefs about one’s ability to determine their own internal states and behavior) [26]. Perceived control is influenced by *perceived susceptibility* (beliefs about the likelihood of getting a disease) [25] and *exposure to risk*. Individual perceptions also include: *attitudes* (evaluative judgments related to an object) [27], *risk sensitivity* (the degree to which individuals respond to a given hazard) [11], *specific fear* (fear-arousing thoughts unique to a given hazard) [11] and *risk denial* (individuals often perceive their own risk as smaller than the risk of other people) [28].

The health belief model (HBM) was used as the theoretical framework because one of its main goals is to change one’s perceptions and behaviors to prevent adverse health outcomes. The HBM consists of various concepts designed to predict whether people will engage in behaviors to prevent, screen for or control diseases [25]. These concepts include susceptibility, seriousness, benefits and barriers to a given behavior, cues to action and self-efficacy [25]. Additionally, various factors including: age, gender, ethnicity, personality, socioeconomics and knowledge often influence perceptions and behavior [25]. A meta-analysis of the effectiveness of HBM variables in predicting health behavior such as smoking cessation and dental care found that perceived benefits and perceived barriers were the strongest predictors of health behavior [29]. Initially the items in the questionnaire were developed to address each of the concepts in the two models, for example the item that was developed to assess risk denial was “I am less likely to fall compared to other people my age.” There were two advisory groups in the development of the items. Physiotherapists with expertise in aging and mobility research (*n* = 6) reviewed the RPQ and provided feedback about the content; older adults (n = 6) also provided feedback about comprehension and wording. Each item in the questionnaire was reviewed with an older adult asking them what they thought the question was asking to assess the face validity of each item within the questionnaire. The older adults were asked to suggest wording for the item if they did not think it was clear. The feedback from the older persons was reviewed by the development group who were all physiotherapists and physiotherapy students in their final term and used the feedback from both the older adults and the physiotherapists to make changes to the items and then reviewed them again with both advisory groups.

Following development and revision, the RPQ was tested using a cross-sectional design. Ten community-dwelling older adults, ≥ 65 years, were recruited from an ongoing study where persons were assessed annually for risk of functional decline. Participants were selected based on their balance scores from the Short Physical Performance Battery [30] to reflect a range of balance ability; 3 participants scored ≤2 points (high level of balance impairment), 5 participants scored 3 points (moderate impairment) and 2 participants scored 4 points (low level of impairment); 60% of the participants had fallen in the past year, however, only 40% perceived themselves at risk of falling. Participants’ balance was also measured using the Berg Balance Scale [31] and a very weak correlation was found between participants’ balance and their perceived risk of falling as measured by the Risk Perception Questionnaire (r = 0.02).

The current study addressed the following research questions in older adults with DM2: 1) What is the test-retest reliability of the RPQ (at least two days between test and retest) as measured by an Intraclass Correlation Coefficient? 2) What is the internal consistency of items on the RPQ as measured by Cronbach’s alpha? 3) What is the construct validity of the RPQ as measured by its correlation with the Fall Efficacy Scale-International? 4) What is the factor structure of the RPQ determined by exploratory factor analysis.

## Methods

The STROBE reporting guidelines were used for this study [32].

This was a prospective cohort study. The convenience sample included people ≥55 years, with a self-reported diagnosis of DM2, living in the community and able to follow verbal instructions in English. The RPQ was initially developed and tested using community-dwelling older adults (≥ 65 years). For the present study, community-dwelling older adults (≥ 55 years) with type 2 diabetes were recruited; 55 years was chosen as the minimum age because individuals with diabetes begin to experience functional decline earlier than older adults without diabetes due to diabetes-related complications (e.g. peripheral neuropathy). Participants were recruited from various exercise programs across Hamilton, Ontario (*n* = 14) and a newspaper advertisement (*n* = 16). Assessments were conducted from May, 2015 to July, 2016 at McMaster University and outpatient settings across Hamilton Ontario. Ethics approval was obtained from the Hamilton Integrated Research Ethics Board (HIREB), REB #: 15–346-S.

### Outcome measures

#### Risk perception questionnaire

The RPQ consists of 20 items in 5 subscales: risk-perception of falling, risk factors, internal/external factors, individual perceptions and self-efficacy. Each item is rated on a 7-point Likert scale from strongly disagree to strongly agree. Individuals’ scores from each of the 5 subscales are added to produce an overall score out of 140. Higher scores on the RPQ indicate higher perceived risk of falling (see text file, Additional file 1 Digital Content 1, which shows the full RPQ).

#### Falls efficacy scale- international

The Falls Efficacy Scale- International (FES-I) is a 16-item tool used to assess fear of falling in older adults during physical and social activities [33]. Fear of falling is assessed on a 4-point scale: 1-not at all concerned about falling to 4-very concerned about falling [33]. Total scores range from 16 to 64, a cut point of 23 points is used to differentiate between individuals with low concern (16–22) and high concern (23–64) about falling [34]. With this suggested cut point, the sensitivity and specificity of the FES-I are 90.9 and 47.2% respectively (gold standard was serum parathyroid levels) [34]. The FES-I has demonstrated high test-retest reliability, ICC = 0.96, and high internal consistency, Cronbach’s alpha = 0.96 in older adults with or without a history of fear of falling [34].

### Rapid assessment of physical activity (RAPA)

The RAPA assesses physical activity, strength and flexibility [35]. It contains statements about physical activities which increase in amount and intensity; individuals answer yes if a statement accurately describes their physical activity [35]. The RAPA is scored by choosing the highest statement (out of 7) with a ‘yes’ response [35]. The last two items on the RAPA assess strength and flexibility and are scored separately [35]. Criterion validity of the RAPA, measured against the Community Healthy Activities Model Program for Seniors (CHAMPS), revealed a moderate correlation between the RAPA and the CHAMPS, r = 0.54, *p* < 0.001 and the sensitivity and specificity of the RAPA were 81 and 69% respectively [35].

### Procedures

**Fig 2:**
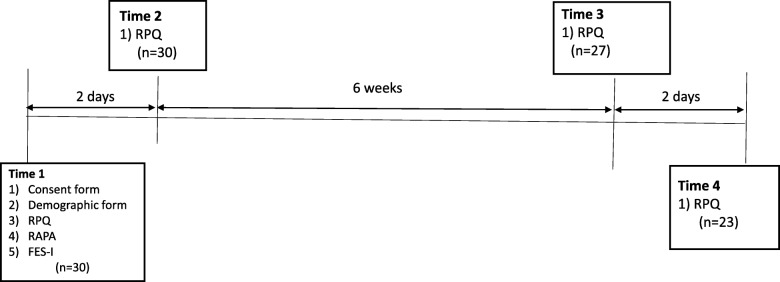
Timeline of assessments

Participants were tested at 4-time points (see Fig. 2) At time 1 (T1) (baseline) the RPQ, RAPA and FES-I were administered. At time 2 (T2), at least two days later, [36] the RPQ was administered again to assess its test-retest reliability. The RPQ was also administered at time 3 (approximately 6 weeks later) and time 4 (at least 2 days after T3) to determine its test-retest reliability when administered by phone.

### Sample size calculation

A sample size of 30 participants was determined based on the following parameters: hypothesized ICC = 0.80, type I error = 0.05, type II = 0.20. After adjusting for an attrition rate of 25%, a minimum of 40 participants was needed (see text file, Additional file 2 Digital Content 2, which shows the full sample size calculation).

### Statistical analyses

Statistical analyses were performed in STATA version 13 for Windows (Stata Statistical Software: Release 13). Descriptive statistics were performed for demographic variables. Means and standard deviations were calculated for normally distributed data while medians and 1st and 3rd quartiles were calculated for data that were not normally distributed.

### Missing data

We examined the missing data in our study to assess if the missing values were missing completely at random (MCAR) which refers to missing data that does not depend on the dependent variable, the covariates or the study design [37]. Little’s MCAR test showed that our data were missing completely at random (χ^2^ = 16.88, DF = 20, *p* = 0.66). The amount of missing data differed at each time point. At T1 and T2 there was no missing data. At T3 there was 10% missing data and at T4 there was 23.3% missing data. Overall, across all time points, there was 8.3% missing data. Missing data analysis was performed in SPSS version 25 (IBM SPSS Statistics for Windows, Version 25.0); missing data was imputed using the expectation-maximization method [38].

### Test-retest reliability

Test-retest reliability of the RPQ was measured in person and by phone. A two-way random effects model was used to calculate the intraclass correlation coefficient (ICC 2, 1). A two-way random effects model assumes that random error comes from both the raters and the participants [39].

Test-retest reliability was assessed by calculating the intraclass correlation coefficient (ICC). The ICC is defined as follows: between subjects variability ÷ (between subjects variability + error); as the error term decreases the ICC moves from 0 to 1 indicating perfect reliability [39]. There is no universal consensus for how the magnitude of the ICC should be interpreted; Fleiss (as cited in Oremus et al. 2012) proposed a classification for the strength of test-retest reliability based on the ICC as follows: < 0.40 poor, 0.40–0.75 fair to good and > 0.75 excellent [40]. Absolute reliability refers to the degree to which repeated measurements of the same instrument on the same individual vary around the true score, the smaller the variation in repeated measurements the higher the absolute reliability [41]. Absolute reliability was measured by the standard error of measurement (SEM). The SEM and ICC of a test are inversely related; if a test has perfect reliability, ICC = 1.0, then the SEM will be zero indicating no error of measurement [42].

### Bland-Altman method

We used the Bland-Altman method to determine the agreement between RPQ scores at time 1 and time 2 (in person administration) as well as time 3 and time 4 (phone administration). The Bland-Altman method uses a plot to describe the agreement between two quantitative measurements and quantifies this agreement by constructing limits of agreement [43]. The limits of agreement are calculated using the mean and the standard deviation of the differences between the two measurements; the upper limit of agreement = mean difference + (standard deviation of the difference × 1.96), the lower limit of agreement = mean difference - (standard deviation of the difference × 1.96) [43]. The Y axis of the Bland-Altman plot represents the difference between paired measurements while the X axis represents the mean of the paired measurements [43]. According to this method, all data points should lie within ± 2 standard deviations of the mean difference [43].

### Internal consistency

Internal consistency assesses the degree to which items on a test are interrelated [44]. Internal consistency was measured using Cronbach’s alpha. Alpha varies from 0 to 1, high alpha values indicate a high degree of interrelatedness among items on a test [44]. Alpha values between 0.70–0.95 are considered good, however it is important to note that alpha is influenced by the number of items on a test; the more items on a test the higher alpha will be, and caution should be taken when interpreting alpha values > 0.90 because this may indicate the presence of redundant items [44]. We calculated the internal consistency of the entire RPQ and each subscale.

### Construct validity

Construct validity of the RPQ was assessed by determining its correlation with the FES-I using Pearson’s correlation coefficient. Persons who score high on the FES-I should also score high on the RPQ. Recommendations for interpreting the strength of a correlation between two constructs suggest the following: r = 0–0.19 a very weak correlation, r = 0.20–0.39 weak correlation, r = 0.40–0.59 moderate correlation, r = 0.60–0.79 strong correlation and r = 0.80–1 very strong correlation [45]. RPQ total scores were normally distributed while FES-I total scores were positively skewed. Therefore, we performed a logarithmic transformation to the FES-I scores before Pearson’s correlation coefficient was calculated.

### Exploratory factor analysis

Exploratory factor analysis (EFA) was performed to determine how the items on the RPQ correlated with each other and to determine whether the items could be grouped into subscales. A preliminary Kaiser-Meyer-Olkin (KMO) test was performed to determine sampling adequacy, datasets with KMO > 0.5 are considered acceptable for EFA [46].

## Results

### Participant characteristics

Thirty older adults with DM2 were enrolled; 60.0% were female (Table 1). The mean (standard deviation) age and duration of type 2 diabetes mellitus were 68.6 (6.9) years and 13.2 (8.2) years respectively. The median number of falls in the last 12 months was 1 (1st quartile: 0, 3rd quartile: 2) (Table 1). On average, participants reported a high fear of falling, median FES-I score 25 (Table 1). Twenty participants (66.7%) reported sensory changes which manifested as nerve pain in fingers and toes, 6 participants (20.0%) reported diabetic peripheral neuropathy, 1 participant (3.3%) reported diabetic retinopathy and 2 participants (6.7%) reported diabetic foot ulcers (Table 1). On average, participants reported 2.3 comorbid conditions. Scores from the RAPA indicated that most participants were under active (60.0%) (Table 2a). Most participants reported that they engage in strength and/or flexibility training (Table 3).

**Table 1:** Demographic characteristics of participants

Participant Characteristics	All participants (*N* = 30)
Age, Mean (SD)	68.6 (6.9)
Female	18 (60.0%)
Duration of diabetes in years, Mean (SD)	13.2 (8.2)
Falls Efficacy Scale – International score, Median (1st, 3rd quartiles)	25 (22–37)
Falls in the past year, n (%)	
0	14 (46.7%)
1	5 (16.7%)
2	7 (23.3%)
3 or more	4 (13.3%)
Nerve pain in the extremities, n (%)	20 (66.7%)
Diabetes related complications, n (%)	
Diabetic peripheral neuropathy	6 (20.0%)
Diabetic retinopathy	1 (3.3%)
Autonomic neuropathy	0 (0.0%)
Diabetic foot ulcers, n (%)	2 (6.7%)

**Table 2:** A Physical activity levels based on the scores from the Rapid Assessment of Physical Activity (RAPA)

Physical activity level	Frequency (%)
Sedentary	0 (0.0%)
Under-active	2 (6.7%)
Under-active regular (light activities)	5 (16.7%)
Under-active regular (moderate activities)	10 (33.3%)
Under-active regular (vigorous activities)	1 (3.3%)
Active (moderate activities)	10 (33.3%)
Active (vigorous activities)	2 (6.7%)

**Table 3:** B Strength and flexibility obtained from the Rapid Assessment of Physical Activity (RAPA)

RAPA score	Description	Frequency (%)
0	No strength/flexibility training	5 (16.7%)
1	Strength training only	7 (23.3%)
2	Flexibility training only	7(23.3%)
3	Strength and flexibility training	11 (36.7%)

### Missing data

At time 1 and time 2, no persons were lost to follow-up (0% missing data). At time 3, three participants were lost to follow-up because our attempts to contact them were not successful (10.0% missing data). At time 4, three more participants were lost to follow-up because our attempts to contact them were not successful and one person did not want to continue the study (23.3% missing data).

### Test-retest reliability

**Fig. 3:**
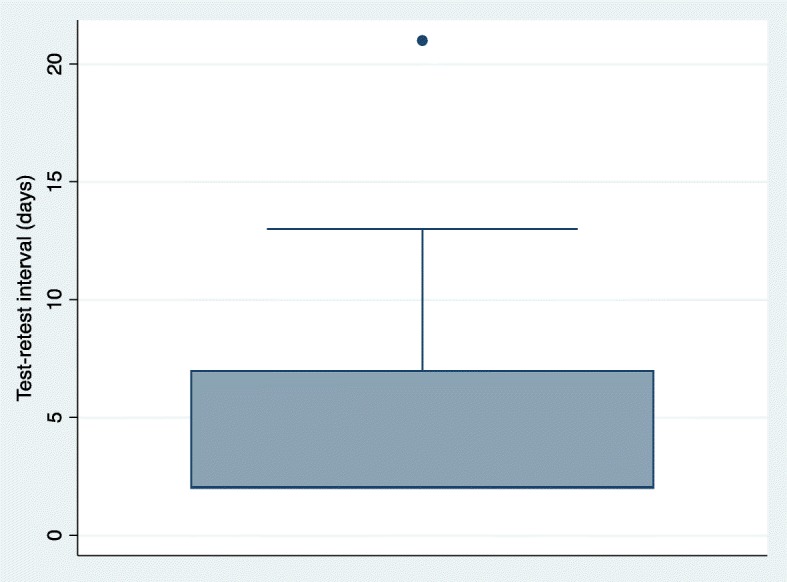
Box plot of test-retest interval in days for in-person administration of the Risk Perception Questionnaire

**Fig. 4:**
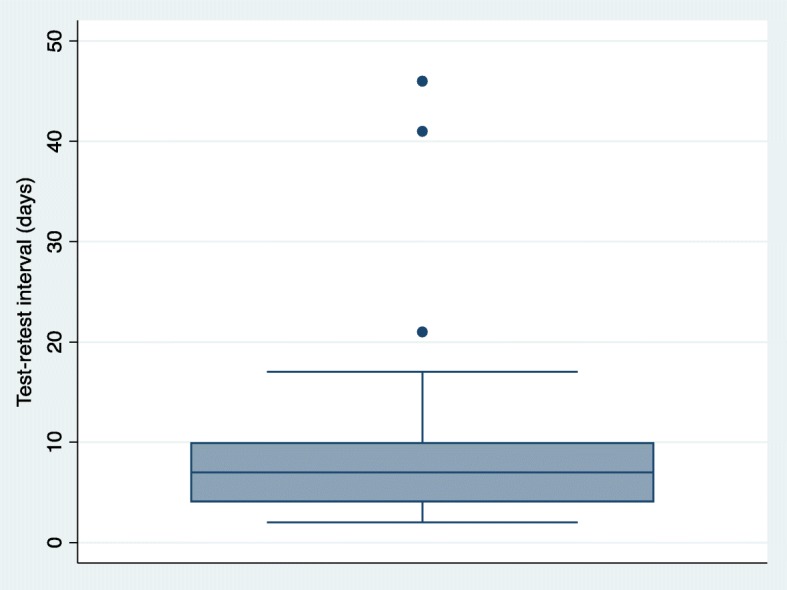
Box plot of test-retest interval in days for phone administration of the Risk Perception Questionnaire

For time 1 and time 2, 28 participants (93.3%) had a test-retest interval between 2 and 7 days and 2 participants (6.7%) had a test-retest interval of greater than 7 days (13 and 21 days respectively). The ICC for the RPQ when administered in person was ICC = 0.78, 95% Confidence Interval, CI: 0.59–0.89, *p* < 0.001. The standard error of measurement (SEM) of the RPQ was 7.06. The distribution of difference scores between test and retest were consistent with a normal distribution (determined by the Shapiro-Wilks test). No participants were lost to follow-up at T1 and T2. For time 3 and time 4, 12 participants (40.0%) had a test-retest interval between 2 and 7 days, 11 participants (36.7%) had a test-retest interval of greater than 7 days (8, 10, 17, 21, 41 and 46 days respectively) and 7 participants (23.3%) were lost to follow-up. Following data imputation (expectation maximization), the ICC (95% CI) of the RPQ when administered by phone was ICC = 0.82, 95% CI: 0.65–0.91, *p* < 0.001. The standard error of measurement (SEM) of the RPQ was 5.93. The distribution of difference scores between test and retest were consistent with a normal distribution (determined by the Shapiro-Wilks test). We also conducted a sensitivity analysis with only persons who completed T3 and T4 (no imputation), ICC = 0.82, 95% CI: 0.62–0.92, p < 0.001 and the SEM = 6.45. Lastly, we conducted sensitivity analyses for in-person (T1-T2) and phone (T3-T4) administration of the RPQ by removing participants who had a very large test-retest interval (see Figs. 3 and 4 for outliers). Sensitivity analysis for in-person administration of the RPQ: ICC = 0.79, 95% CI: 0.59–0.89 and the SEM = 6.99. Sensitivity analysis for the phone administration of the RPQ: ICC = 0.83, 95% CI: 0.62–0.93 and the SEM = 6.33.

### Bland-Altman results

**Fig 5:**
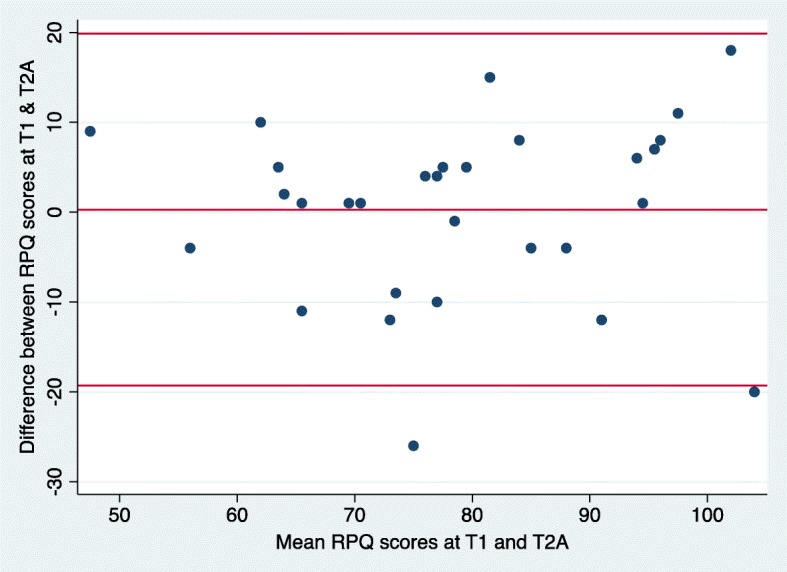
Bland-Altman plots for RPQ scores at time 1 and time 2. The middle line represents the mean difference between RPQ scores at time 1 and time 2. The lower and upper lines represent the upper and lower 95% confidence limits respectively

**Fig 6:**
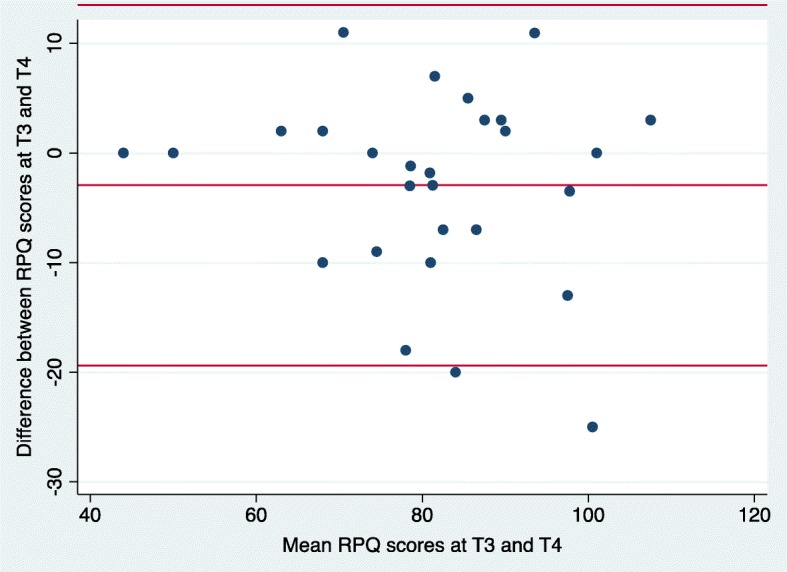
Bland-Altman plots for RPQ scores at time 3 and time 4. The middle line represents the mean difference between RPQ scores at time 3 and time 4. The lower and upper lines represent the upper and lower 95% confidence limits respectively

The mean difference in RPQ scores between time 1 and time 2 was 0.27 (− 3.46, 4.0) which was not significantly different from zero (t = 0.15, *p* = 0.88). This indicates that on average, the RPQ produced the same scores at time 1 and time 2, thus indicating good agreement between RPQ scores at time 1 and time 2. The standard deviation of the difference was 9.9. The 95% limits of agreement were − 19.31 to 19.85 (Fig. 5). The mean difference in RPQ scores between time 3 and time 4 was − 2.95 (− 6.08, 0.19); this mean difference was not significantly different from zero, t = − 1.92, *p* = 0.06. This indicates that on average, the RPQ produced the same scores at time 3 and time 4, thus indicating good agreement between RPQ scores at time 3 and time 4. The standard deviation of the difference was 8.39; 95% limits of agreement were − 19.39 to 13.49 (Fig. 6).

### Internal consistency

The internal consistency of the entire questionnaire was good, alpha = 0.78. The self-efficacy subscale had the highest internal consistency, alpha = 0.79 followed by the internal/external factors subscale, alpha = 0.64. The individual perceptions subscale and the risk factors subscale had the lowest internal consistencies, alpha = 0.60 and alpha = 0.63 respectively.

### Construct validity

Construct validity of the RPQ was assessed by determining its correlation with the FES-I using Pearson’s correlation coefficient. There was a moderate correlation between the RPQ and the FES-I, r = 0.52 (0.20–0.74), *p* = 0.003. We therefore assume that the RPQ has adequate construct validity.

### Exploratory factor analysis (EFA)

The Kaiser-Meyer-Olkin (KMO) measure of sampling adequacy was 0.35. Datasets with KMO > 0.50 are considered acceptable for EFA [46]. Therefore, we did not proceed with the EFA.

## Discussion

We explored the test-retest reliability, internal consistency and construct validity of a falls risk perception questionnaire in older adults with DM2. The test-retest reliability of the RPQ was good when administered in person and by phone. The internal consistency of the RPQ was also good. Lastly, scores on the RPQ were moderately correlated with scores on the Falls Efficacy Scale International indicating adequate construct validity.

A test is considered to have excellent test-retest reliability if ICC > 0.75 excellent [40]. Therefore, the RPQ demonstrated excellent test-retest reliability when administered in person and by phone. Our goal was to have a 2-day test-retest interval for the RPQ. However, in the real- world setting, it is difficult to test all participants with the same test-retest interval due to practical implications such as participant availability. Therefore, it is possible that the reliability of the RPQ could have been affected by variability of the test-retest interval. To address this issue, Marx et al. (2003) conducted a study to determine the impact of different test-retest intervals on test-retest reliability (ICC); they compared the reliability of various tests administered with a 2-day test-retest interval versus a 2-week test-retest interval [36]. They found overlapping ICC 95% confidence intervals for both time intervals indicating that the test-retest reliability (ICC) did not differ for 2 days versus 2 weeks [36]. In our study, 96.7% of participants had a test-retest interval between 2 days and 2 weeks for the in-person administration of the RPQ and 78.3% of participants had a test-retest interval between 2 days and 2 weeks for the phone administration of the RPQ. Therefore, given our data and the findings by Marx et al. (2003), we do not believe that the variability of the test-retest interval affected the test-retest reliability of the RPQ.

To examine the impact of missing data, we conducted various sensitivity analyses. The first sensitivity analysis was done with completers only (no imputation); this sensitivity analysis found no change in the ICC or 95% CI (overlapping 95% CI) and an increase in the SEM compared to the original analysis with the imputed data. For the second sensitivity analysis, we removed outliers (i.e. persons with very long test-retest interval, as determined by box plots). This sensitivity analysis showed no change in the ICC (same 95% CI) and a slight decrease in the SEM for the in-person administration. For the phone administration, the sensitivity analysis showed no change in ICC (overlapping 95% CI) and a slight increase in the SEM. This RPQ also had good test-retest reliability, ICC = 0.66, 95% CI: 0.30–0.85, in women, > 45 years, following a distal radius facture [47]. Overall, the RPQ has demonstrated good test-retest reliability in research settings thus far. The RPQ scores also showed good agreement based on the Bland-Altman analysis for both methods of administration. For internal consistency, acceptable values for Cronbach’s alpha range from 0.70–0.95 [44]. However, extremely high alpha values (> 0.90) may indicate the presence of redundant items [44]. We conclude that the internal consistency of the RPQ was good (Cronbach’s alpha = 0.78). We attempted to conduct exploratory factor analysis but our KMO measure of sampling adequacy was 0.35, therefore we could not proceed any further. Sample size is the main factor that affects sampling adequacy [48]. In our study, sampling adequacy (KMO) was low due to small sample size (*n* = 30). In general, sample size ≥200 is considered large enough to perform EFA [48]. Therefore, future studies should be done with larger samples to examine the factor structure of the RPQ using EFA. Lastly, there was a moderate positive correlation between scores on the RPQ and scores on the FES-I, r = 0.52 (0.20–0.74), *p* = 0.003. Perceived risk of falling and fear of falling share some constructs; for example, self-efficacy is considered an important construct of both concepts [49]. However, there is an important difference between them; fear of falling has a large emotional aspect whereas perceived risk of falling is largely evaluative/judgment based. For example, fear of falling (FOF) refers to a persistent concern or worry about falling which causes the individual to restrict activities of daily living [50]. On the other hand, perceived risk of falling is a multi-dimensional concept that considers awareness and judgements about the probability of an outcome (e.g. a fall) and its potential consequences as well as judgments about the importance of the risk to the individual [5]. Additionally, perceived risk is based on a broad range of factors (e.g. knowledge of risk factors [10], physical environment [19] and culture [21]) which can be used to target older adults’ behavior in order to mitigate their risk of falling compared to fear of falling which is primarily based on emotions (e.g. worry, fear and concern) [50].

Falls risk assessment is an important part of clinical care for older adults. Balance is assessed using standardized tests such as the Tinetti Balance and Gait Test and the Berg Balance Test [51]. These tests examine impairments in gait and balance that may predispose older adults to falling [51]. Balance in older adults is well understood however, perceived risk of falling is not [52]. Perceived risk of falling influences individuals’ willingness to engage in fall prevention behaviors [52]. A moderate level of perceived risk is considered optimal; this causes the individual to exercise some caution without restricting their activity altogether [52, 53]. The risk perception literature suggests that perceived risk shapes health behavior. A meta-analysis of 34 studies demonstrated that individuals with higher perceived illness likelihood, susceptibility and severity were significantly more likely to get vaccinated [12]. The Health Action Process Approach states that risk perception itself is not sufficient for the formation of behavioral intentions, rather it helps initiate the contemplation and elaboration of thoughts, consequences and competencies needed to start behavior change [14]. Perceived self-efficacy is another factor that mediates the relationship between perceived risk and health behavior. Rimal (2001) measured perceived risk of Cardiovascular Diseases (CVDs) by asking participants to rate both their likelihood of acquiring CVDs and CVD-related self-efficacy (their confidence in their ability to engage in CVD-prevention behaviors such as exercising regularly and consuming a healthy diet) [54]. Rimal (2001) identified four distinct groups of individuals: responsive (high perceived risk + high self-efficacy), proactive (low perceived risk + high self-efficacy), avoidant (high perceived risk + low self-efficacy) and indifferent (low perceived risk + low self-efficacy) [54]. Individuals classified as responsive or proactive were significantly more likely to think about and use CVD-related information than individuals classified as avoidant or indifferent [54]. These findings suggest that when trying to promote healthy behavior both perceived risk and self-efficacy should be examined.

## Limitations

There were several limitations. For example, to increase the generalizability of our results, we intended to recruit equal numbers of older adults from different age cohorts however, this was not achieved. Most of our participants were 66–75 years (50%), while only 36.67% of participants were 55–65 years, 10% of participants were 76–85 years. Therefore, our results may be more applicable to the two younger cohorts and less applicable to the two older cohorts. Additionally, our sample size calculation showed that 30 participants were needed (no attrition), therefore our sample was adequately powered for the in-person reliability due to no attrition. In contrast, 3 participants were lost to follow-up at T3 and 4 participants were lost to follow-up at T4 which reduced the power of our calculations for phone reliability. However, missing data was addressed using imputation and sensitivity analyses which showed no difference in results with and without the missing data. Further psychometric testing of this questionnaire should be done using sample sizes that are adequate to compensate for attrition.

## Conclusion

The RPQ is currently the only instrument designed to measure perceived risk of falling in older adults. This is the first time that perceived risk of falling has been measured in older adults with DM2. Physical function of older adults with an increased risk of falling due to chronic conditions such as DM2 should be closely monitored. Clinicians should assess perceived risk of falling in addition to balance to gain a more comprehensive understanding of overall risk of falling. The RPQ has demonstrated good test-retest reliability, good internal consistency and adequate construct validity in older adults with DM2. Further testing should be done in different patient populations using larger sample sizes.

## Supplementary information

**Additional file1.** Digital Content 1. Risk Perception Questionnaire.

**Additional file 2.** Digital Content 2. Sample size calculation.

## Abbreviations

ADLs: Activities of daily living
DM2: Type 2 Diabetes Mellitus
FES-I: Falls Efficacy Scale- International
HBM: Health belief model
IADLs: Instrumental activities of daily living
ICC: Intraclass Correlation Coefficient
RAPA: Rapid assessment of physical activity
RPQ: Risk Perception Questionnaire
