# Supplementary material for: Test-retest reliability, internal consistency, construct validity and factor structure of a falls risk perception questionnaire in older adults with type 2 diabetes mellitus: a prospective cohort study
Source: Arch Physiother. 2019 Dec 2;9:14. doi: 10.1186/s40945-019-0065-4 (PMC6886215; doi:10.1186/s40945-019-0065-4)
Supplement: Supplementary file 1 — Additional file1. Digital Content 1. Risk Perception Questionnaire. [file 40945_2019_65_MOESM1_ESM.pdf]

McMaster University

Risk Perception Questionnaire

Please respond to the following questions to the best of your ability. Please indicate your response to each question by circling the most appropriate number on the scale that follows each question.

Risk perception of falling

1. I feel that I am at risk for having a fall

1 2 3 4 5 6 7

strongly disagree strongly agree

Risk factors

Please rate if you feel the following factors may increase your risk for falling.

2. A fall in the past year

1 2 3 4 5 6 7

strongly disagree strongly agree

Please rate if you feel the following factor may increase your risk for falling.

3. Weakness in your legs

1 2 3 4 5 6 7

strongly disagree strongly agree

Please rate if you feel the following factor may increase your risk for falling.

4. Difficulty moving around the house or community

1 2 3 4 5 6 7

strongly disagree strongly agree

Please rate if you feel the following factor may increase your risk for falling.

5. Using a cane or walker

1 2 3 4 5 6 7

strongly disagree strongly agree

Please rate if you feel the following factor may increase your risk for falling.

6. Being physically active (beyond your regular daily activity)

1 2 3 4 5 6 7

strongly disagree strongly agree

Internal/external factors

I believe that my risk of falling is influenced by:

7. Information given to me by my friends and family

1 2 3 4 5 6 7

strongly disagree strongly agree

I believe that my risk of falling is influenced by:

8. Information given to me by the media (i.e. TV, radio, newspaper, etc.)

1 2 3 4 5 6 7

strongly disagree strongly agree

I believe that my risk of falling is influenced by:

9. Information given to me by my doctor, physiotherapist or health care worker

1 2 3 4 5 6 7

strongly disagree strongly agree

I believe that my risk of falling is influenced by:

10. My cultural and religious beliefs

1 2 3 4 5 6 7

strongly disagree strongly agree

11. I feel that getting older increases my risk of falling

1 2 3 4 5 6 7

strongly disagree strongly agree

Individual perceptions

12. a. I feel that people my age are more likely to fall than people who are younger

1 2 3 4 5 6 7

strongly disagree strongly agree

b. I am less likely to fall when compared to other people my age

1 2 3 4 5 6 7

strongly disagree strongly agree

13. I feel that I am able to protect myself from falling

1 2 3 4 5 6 7

strongly disagree strongly agree

14. If I had a fall, it would result in serious consequences

1 2 3 4 5 6 7

strongly disagree strongly agree

15. My attitude about aging influences my risk of falling

1 2 3 4 5 6 7

strongly disagree strongly agree

16. Avoiding a fall is just as important as my other health concerns

1 2 3 4 5 6 7

strongly disagree strongly agree

Self-efficacy

17. I feel confident that I can maintain my balance when I walk outside in the community

1 2 3 4 5 6 7

strongly disagree strongly agree

18. I feel confident that I will not fall when walking outside in poor weather

1 2 3 4 5 6 7

strongly disagree strongly agree

19. I feel confident that I will not fall when moving around in my house

1	2	3	4	5	6	7
strongly disagree					strongly agree	

Thank you for taking the time to complete the questionnaire. Please return it to your health care provider.

/7	Risk perception of falling
/35	Risk factors
/35	Internal/external factors
/42	Individual perceptions
/21	Self-efficacy
/140	Total score
