# Supplementary material for: Test-retest reliability, internal consistency, construct validity and factor structure of a falls risk perception questionnaire in older adults with type 2 diabetes mellitus: a prospective cohort study
Source: Arch Physiother. 2019 Dec 2;9:14. doi: 10.1186/s40945-019-0065-4 (PMC6886215; doi:10.1186/s40945-019-0065-4)
Supplement: Supplementary file 2 — Additional file 2. Digital Content 2. Sample size calculation. [file 40945_2019_65_MOESM2_ESM.pdf]

Sample size calculation

Parameters:

- Type I error = 0.05
- Type II error = 0.20
- k = 2
- hypothesized ICC = 0.80

n = $\frac{0.5\ k\ (Z\alpha+Z\beta)^\wedge 2}{\sigma 2\ (k-1)}$ + 2

$Z\alpha$ = 1.645

$Z\beta$ = 0.842

$R_{expected}$ = 0.80

$Z_{Rexpected}$ = 0.5 natural log $\frac{1+(k-1)Rexpected}{1-Rexpected}$

$Z_{Rexpected}$ = 1.0986

$R_{lowerlimit}$ = 0.80 – 0.10 = 0.70

Z_{Rnull} = 0.5 natural log $\frac{1+(2-1)0.70}{1-0.70}$

Z_{Rnull} = 0.867

$\sigma^2 = Z_{Rexpected} - Z_{Rnull}$

σ^2 = 0.2316

$$n = \frac{0.5\ k\ (Z\alpha + Z\beta)^{\wedge}2}{\sigma 2\ (k-1)} + 2$$

n = 28.706

Sample size = 29 participants round to 30 participants

Sample size adjusted for attrition rate of 25% = 30 ÷ (1-0.25) = 40

Final sample size = 40 participants
